# Optimization of γ-Aminobutyric Acid (GABA) Accumulation in Germinating Adzuki Beans (*Vigna angularis*) by Vacuum Treatment and Monosodium Glutamate, and the Molecular Mechanisms

**DOI:** 10.3389/fnut.2021.693862

**Published:** 2021-09-09

**Authors:** Xiujie Jiang, Qingpeng Xu, Aiwu Zhang, Yong Liu, Liqin Zhao, Liwei Gu, Jianbin Yuan, Hongdou Jia, Xinting Shen, Zhijiang Li, Dongmei Cao, Dongjie Zhang

**Affiliations:** ^1^National Coarse Cereals Engineering Research Center, Heilongjiang Bayi Agricultural University, Daqing, China; ^2^College of Food Science, Heilongjiang Bayi Agricultural University, Daqing, China; ^3^Experimental Equipment Management Center, Heilongjiang Bayi Agricultural University, Daqing, China

**Keywords:** γ-aminobutyric acid, vacuum, MSG, adzuki bean, germination

## Abstract

This study aimed to investigate the optimal hypoxic and monosodium glutamate (MSG) stress conditions for the enrichment of γ-Aminobutyric acid (GABA) in germinating adzuki beans and to reveal the potential underlying molecular mechanisms of GABA accumulation. Using single-factor experiments and response surface model, we investigated the effects of germination time, germination temperature, vacuum time, and MSG concentration on GABA contents, and further explored the activity and gene expression of glutamate decarboxylase (GAD) and polyamine oxidase (PAO) critical rate restriction enzymes during GABA synthesis. The optimal soaking temperature, soaking time, and pH conditions were 35°C, 16 h, and 5, respectively. Furthermore, the optimal germination conditions for optimal GABA enrichment were 48 h, 1.99 mg/ml MSG concentration, germination temperature of 31.49°C, and vacuum time of 15.83 h. Under such conditions, the predicted GABA concentration was 443.57 ± 7.18 mg/100 g, with no significant difference between the predicted and experimental data. The vacuum + MSG (FZM) treatment has a maximum contribution rate of GABA to 38.29%, which significantly increase GABA content, and the increase was associated with increased GAD and PAO activity. In addition, MSG in combination with vacuum treatment could significantly induce *VaGAD4* and *VaGAD6* genes in 2 days germination of adzuki beans. According to the results of the present study, vacuum + MSG treatment is an effective approach to enhancing GABA accumulation in germinating adzuki beans, which could be employed in enhancing the functional quality of germinating adzuki beans.

## Introduction

γ-Aminobutyric acid (GABA) is a four-carbon non-proteinogenic amino acid that acts as a major inhibitory neurotransmitter in the central nervous system (1). It participates in a variety of physiological activities in higher animals, such as regulating blood pressure and heart rate and alleviating pain and stress (2, 3). In addition, recent research has revealed that GABA could induce α-cell-mediated β-like cell neogenesis *in vivo*, which presents new prospects for diabetes treatment (4). Due to the potential benefits, the development of foods enriched with GABA has attracted considerable attention.

γ -Aminobutyric acid is extensively distributed in plants, especially in legume species, although its concentration is often low (5). Germination can activate endogenous enzyme activity, inducing complex physiological reactions in plant seeds, leading to the decomposition of protein and macromolecular substances into polypeptides and amino acids, which enhance the nutritional quality and enrich bioactive compounds, especially GABA contents (6, 7). GABA is produced mainly from glutamate (Glu) *via* the catalysis of glutamate decarboxylase (GAD). Afterward, GABA transaminase (GABA-T) catalyzes GABA to undergo a reversible reaction to produce succinic acid semialdehyde, which is then converted into succinic acid that participates in the subsequent tricarboxylic acid cycle (8). The metabolic pathway is known as GABA shunt. GABA can also be enriched through the polyamine degradation pathway, and diamine oxidase (DAO) and 4-aminobutyraldehyde dehydrogenase (ABALDH) are the key enzymes involved in the pathway (9, 10).

Numerous studies have suggested that the combination of germination and hypoxic treatment would improve the GABA contents; for example, Ding et al. observed that GABA increased significantly in the hypoxia-germinated rice (11). In another study, GAD and DAO activity, as well as GABA contents, increased during germination under hypoxia within 24 h (12). Such studies show that germination under hypoxic conditions is a potential strategy for the enrichment of GABA contents in plants.

The adzuki bean, which has been planted in China for more than 2,000 years, has high nutritional value and is a great source of protein, carbohydrates, vitamins, and minerals (13). It also contains diverse amino acids, with a glutamic acid content of 3,608 mg/100 g (14). Monosodium glutamate (MSG) is a prerequisite for GABA synthesis. GABA contents in adzuki bean could also be enriched following germination under environmental stress. Numerous studies have investigated GABA accumulation in soybean (9, 12, 15), fava bean (16, 17), foxtail millet (18), brown rice (19), and buckwheat (20) under stress. Few studies have examined GABA accumulation in adzuki beans during germination under hypoxic conditions, and the underlying mechanisms of GABA enrichment under such conditions remain unknown.

In this study, we investigated the effects of vacuum conditions and MSG treatment on GABA contents in the germinating adzuki beans, in addition to the activity and expression of key enzymes (e.g., GAD and polyamine oxidase, PAO) in the GABA synthesis pathway. The present study aimed to reveal the contribution of exogenous MSG treatment under vacuum stress on GABA synthesis in germinating adzuki bean, and the underlying molecular mechanisms.

## Materials and Methods

### Materials and Chemicals

For this study, adzuki beans (variety: Pearl Red) were purchased from Daqing Ruizefeng Agricultural Technology Co., Ltd (Daqing, China). These adzuki bean seeds were harvested in 2019 and stored at 4°C until use. The GABA standard was purchased from Sigma Aldrich Co. (Shanghai, China). The plant RNA extraction kit (15596018) was purchased from Thermo Fisher Scientific (Shanghai, China), the cDNA synthesis kit (R312-01) was purchased from Vazyme Biotech Co., Ltd (Nanjing, China), and GAD, PAO, and diamine oxidase ELISA kits were purchased from Shanghai Enzyme-linked Biotechnology Co., Ltd. (Shanghai, China). All other reagents were of analytical grade.

### Immersion of Adzuki Beans

An appropriate amount of selected adzuki beans was weighed, rinsed, and the ratio of purified water added to the beans was 1:5. Subsequently, they were disinfected with 0.7% sodium hypochlorite for 15 min. Thereafter, they were water rinsed three times to remove excess sodium hypochlorite, and then soaked in water baths at 15, 20, 25, 30, 35, and 40°C. Citric acid and disodium hydrogen phosphate were used to adjust the pH values of the soaking solutions to 3, 5, 6, 7, 9, and 11, respectively. The soaking time was set to 0, 4, 8, 12, 16, and 20 h. At the end of immersion, adzuki beans were dried and the GABA contents were determined at 645 nm.

### Germination of Adzuki Beans

After soaking the adzuki beans, they were sprinkled evenly in Petri dishes coated with four layers of gauze, soaked in the gauze, and placed in a constant-temperature incubator to germinate. During germination, the samples were dried and the GABA contents determined at different time points. Subsequently, a part of the fresh sample was frozen rapidly using liquid nitrogen and stored in a refrigerator at −80°C for use in the evaluation of other indicators. Each treatment was performed three times.

### Single-Factor Experiment

The effects of germination time, germination temperature, vacuum time, and MSG concentrations on GABA contents in adzuki beans were investigated. The experimental ranges of the four factors were set as follows: the germination time was 12, 24, 36, 48, 60, and 72 h; germination temperature was 15, 20, 25, 30, 35, and 40°C; vacuum time was 4, 8, 12 16, 20, and 24 h; MSG concentrations were 1.0, 1.5, 2.0, 2.5, 3.0, and 3.5 mg/ml. Experiments were performed three times.

### Response Surface Optimization Experiment

Considering the conditions for GABA accumulation during germination were optimized, and according to the Box-Behnken test design, a four-factor three-level response surface experimental design was set up using Design Expert v8.06 (Stat-Ease Inc., Minneapolis, MN, USA). The results were analyzed using ANOVA, regression analysis, and response surface plots (21). The response surface plots were used to determine the optimal GABA formation conditions based on the response surface model (RSM). The experimental factors and levels are listed in Supplementary Table 1.

### GABA Contents Measurement

γ-Aminobutyric acid contents were determined using a slightly improved colorimetric method (22, 23). Germinating adzuki beans (2.5 g) were obtained, after which an appropriate amount of distilled water solution was added. This was followed by dilution to 50 ml and the mixture was leached in a water bath at 30°C to perform ultrasonic extraction for 2 h, 0.5 ml of filtrate filtered, and 0.2 ml of 0.2 mmol/L boric acid buffer (pH 9.0) was added; 1 ml of 6 g/100 ml redistilled phenol solution added, 0.4 ml of NaClO solution with 9% available chlorine content added, shaken well, placed it in a boiling water bath for 10 min, and then immediately placed in an ice-water bath for 20 min with constant shaking. After the solution turned blue-green, 2 ml of 60% ethanol solution was added, flapped again uniformly, left to stand, and then absorbance was measured at 645 nm (UV TU-1810PC; Puxi, Beijing, China). A standard curve was drawn using the absorbance values of standard GABA and used to obtain the regression equation. The GABA contents in adzuki beans were derived using the regression equation and the results were expressed as mg GABA/100 g dry weight.

*y* = 7.4727 *x* – 0.0241 *R*^2^ = 0.9963

### Analysis of the Mechanism of GABA Enrichment

#### Experimental Design

Based on the single-factor experiments and RSM, the germination conditions (germination time, germination temperature, vacuum time, and MSG concentration) influenced GABA production in adzuki beans. Therefore, this section focuses on the mechanisms *via* which vacuum conditions and MSG influenced GABA contents in germinating adzuki beans. The experiments included four treatments, as follows:

(1) Control treatment (CK); (2) 2 mg/ml MSG treatment (FM); (3) 15 h vacuum treatment (FZ); and (4) 15 h vacuum + 2 mg/ml MSG treatment (FZM). The CK soaking conditions were based on the results of the single-factor experiments (soaking time 16 h, soaking temperature 35°C, and pH 5), and germination conditions were based on the surface optimization results (germination temperature 31°C). The FM soaking conditions were similar to those of CK, germination temperature was 31°C, and MSG concentration was 2.0 mg/ml. The FZ soaking conditions were similar to those of CK, germination temperature was 31°C, and vacuum time was 15 h. The FZM soaking conditions were similar to those of CK, germination temperature was 31°C, MSG concentration was 2.0 mg/ml and vacuum time was 15 h. After the treatments were subjected to 2 and 4 days germination, some of the beans were oven-dried and others are frozen using liquid nitrogen for further analyses.

#### Measurement of Enzyme Activity

The samples (5.00 g) were weighed in pre-cooled sterile mortar, and phosphate buffer (pH 6.8) was added to quickly dilute to 50 ml, equilibrated at room temperature for 2 h, centrifuged at 10,000 × *g* for 20 min at 4°C, and the supernatant obtained was crude enzyme solution (24). PAO activity was determined according to ELISA kit instructions, and GAD was determined according to the method of Bartyzel (25).

#### Analysis of GABA Synthase Gene Expression Using Quantitative Real-Time PCR

Sequences of genes encoding *VaGAD* enzyme (*VaGAD1, VaGAD2, VaGAD3, VaGAD4, VaGAD5*, and *VaGAD6*), *VaPAO* enzyme (*VaPAO1, VaPAO2, VaPAO3, VaPAO4, VaPAO5*, and *VaPAO6*) in adzuki beans were obtained from the National Center for Biotechnology Information. The primers were synthesized at TSINGKE Biotechnology Co., Ltd. (Beijing, China). Total RNA was prepared from germinating adzuki beans using a TRIzol™ RNA Kit (15596018, Thermo Fisher Scientific, Waltham, MA, USA). Reverse transcription was performed with 1 μg total RNA using the cDNA synthesis kit (TaKaRa RR047a, Kyoto, Japan) according to the instructions from the manufacturer, and qRT-PCR analysis was performed using SYBR Premix Ex Taq II (TaKaRa, RR820, Dalian, China), and run on a QTOWER quantitative PCR instrument using four channels (Analytik Jena GmbH, qTOWER3G, Germany). The PCR cycling program included an initial denaturation at 95°C for 30 s (40 times), followed by 30 s of extension at 58°C (26). Primer pairs used for quantitative real-time PCR (qRT-PCR) are listed in Supplementary Table 2. At the end of the PCR cycle, the transcript levels of all genes were calculated using the 2^−ΔΔCt^ method (27), and β-actin was used as the internal control gene.

### Statistical Analysis

Each experiment was performed three times, and the data are expressed as mean ± SD. The data were analyzed using IBM SPSS Statistics 20.0 (IBM Corp., Armonk, NY, USA). ANOVA was used to test for significant differences, and Duncan's new multiple range test used to test for significance between the different groups. Line graphs and column graphs were illustrated using MS Excel 2010 (Microsoft Corp., Redmond, WA, US).

## Results

### Effects of Soaking Conditions on γ-Aminobutyric Acid Contents

**Figure 1:**
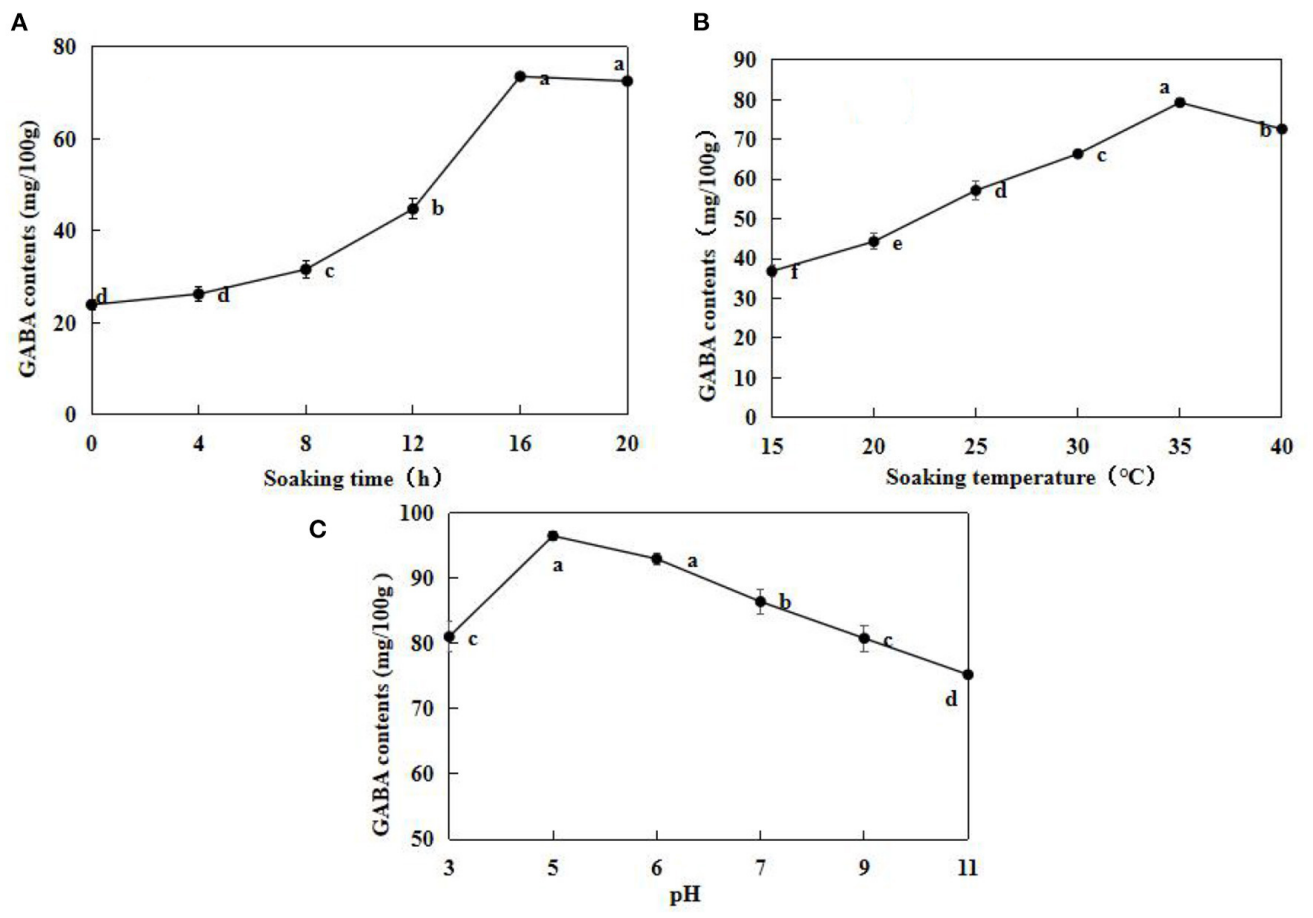
Effects of soaking time **(A)**, soaking temperature **(B)**, and pH **(C)** on adzuki bean GABA contents. The soaking temperature was fixed at 25°C, and the soaking times were 0, 4, 8, 12, 16, and 20 h **(A)**; The adzuki beans were soaked in distilled water at 15, 20, 25, 30, and 35°C for 12 h **(B)**; Soaking temperature was 25°C for 12 h, soaking buffer pH values were 3, 5, 6, 7, 9, and 11. Subsequently, GABA contents in the samples under different conditions were determined. Data are mean ± SD (*n* = 3). Different lowercase letters indicated significant difference at *p* < 0.05.

γ -Aminobutyric acid contents in the un-soaked adzuki beans were very low, at 23.97 ± 1.13 mg/100 g (Figure 1A). The GABA contents were the highest after 16 h, at 73.59 ± 0.66 mg/100 g. After 20 h, the GABA contents decreased by 15% than the contents at 16 h. Therefore, soaking for 16 h was considered to enhance the GABA enrichment in adzuki beans. GABA contents seemed to increase with an increase in soaking temperature. GABA content was highest when the soaking temperature was 35°C (Figure 1B). In addition, GABA content was highest when pH was 5, at 96.53 ± 1.13 mg/100 g (Figure 1C). This indicated that the acidic environment was more conducive for GABA accumulation in adzuki beans. According to single factor analysis results, a soaking time of 16 h, a soaking temperature of 35°C, and pH value of 5 was the optimal soaking conditions.

### Effects of Germination Conditions on γ-Aminobutyric Acid Contents

**Figure 2:**
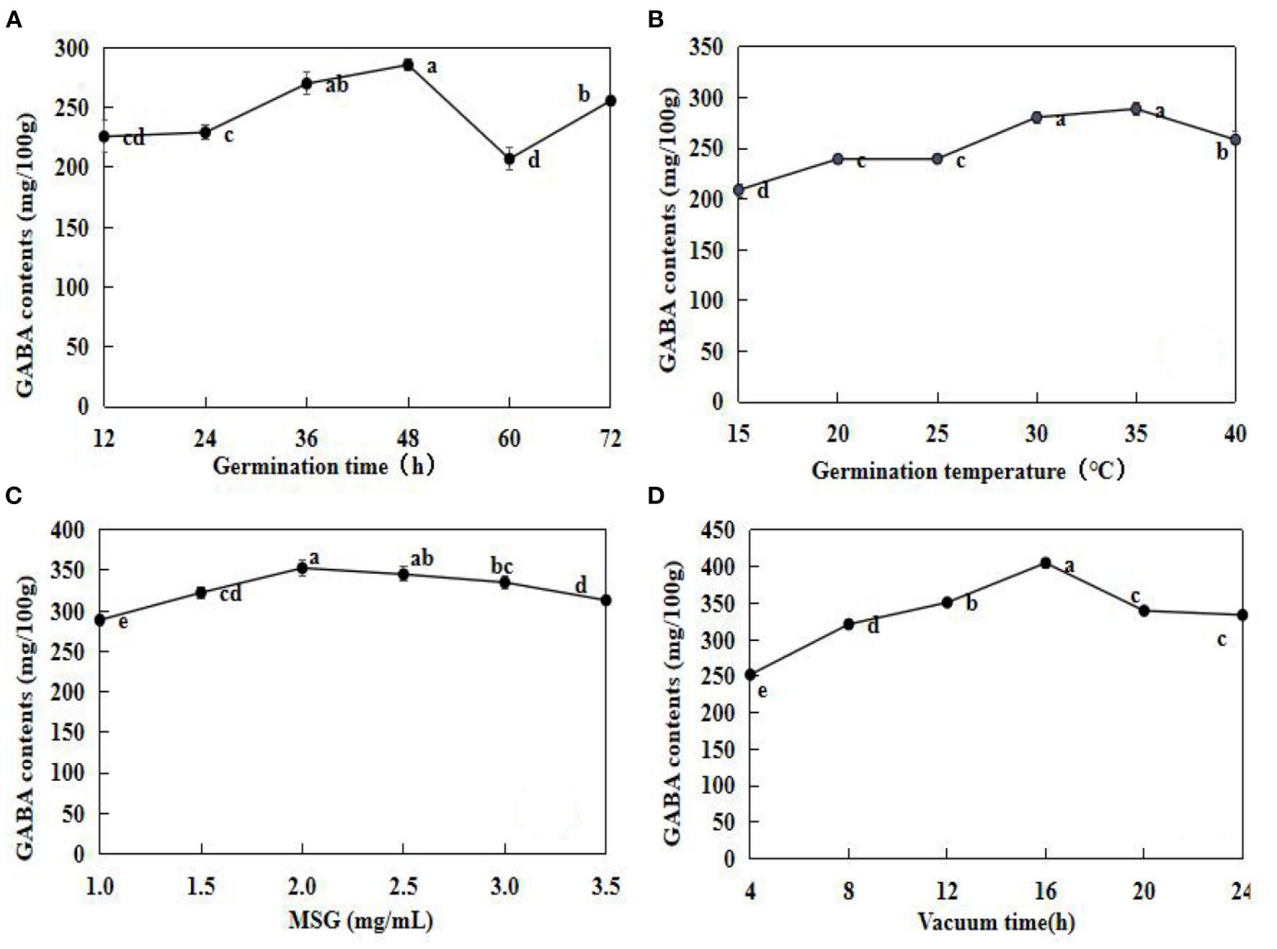
Effects of germination time **(A)**, germination temperature **(B)**, monosodium glutamate (MSG) concentration **(C)**, and vacuum time **(D)** on GABA contents in adzuki bean. After pre- soaking treatments, the beans were subjected to germination experiments. Effect of germination time was tested at 25°C **(A)**; Effect of germination temperature for 24 h **(B)**; Effect of MSG concentration was tested at 25°C and for 24 h **(C)**; Effect of vacuum time was tested 25°C and 24 h **(D)**. The data are mean ± SD (*n* = 3). Different lowercase letters indicate significant differences at *p* < 0.05.

The optimal soaking conditions were applied in the subsequent experiments. The effects of germination time, germination temperature, MSG concentration, and vacuum time on GABA are illustrated in Figure 2.

In general, GABA contents increased with an increase in time, and the highest GABA contents were observed at 48 h (285.55 ± 4.78 mg/100 g) (Figure 2A). However, at 60 h, GABA accumulation decreased by 27.52% when compared with that at 48 h. Similarly, GAD activity remained relatively high at the early stages (0–40 h) of germination, inferred GAD activities were activated during the early germination period (12). The GABA contents were the highest at 35°C, and decreased significantly (*P* < 0.05) at 40°C (Figure 2B). The GABA contents increased gradually in germinating adzuki beans under 1.0–2.0 mg/ml MSG, and then decreased at 2.5 mg/ml, gradually. The GABA contents when treated under vacuum for 4–16 h were the highest at 16 h (405.23 ± 6.39 mg/100 g), and then representing a significant decrease (*p* < 0.05) relative to the concentration at 16 h. According to the results above, both vacuum and MSG treatment could increase GABA contents in germinating adzuki beans, and vacuum treatment had a better effect than MSG.

### Response Surface Optimization of Vacuum and Exogenous MSG on GABA Enrichment

The response surface optimization design factors, levels and results are shown in Table 1, the response surface results were analyzed using Design Expert10 software, and the results of ANOVA are presented in Table 2. The total model was extremely significant (*p* < 0.01), and the lack of fit was not significant (*p* > 0.05). These values indicated that the accuracy of the polynomial model was acceptable, and it was reliable, and it could predict the process parameters of vacuum time and MSG enrichment on GABA contents in adzuki beans. According to the absolute value of the coefficient of the primary term in the binary regression equation, the primary and secondary order of determining the influence of each factor on the response value was germination time (A) > vacuum time (D) > MSG concentration (B) > germination temperature (C).

**Table 1:** Response surface design and results.

**Serial number**	**Factors**				**GABA contents (mg/100 g)**
	**A**	**B**	**C**	**D**	
1	1	1	0	0	392.34 ± 5.78
2	0	−1	0	−1	364.55 ± 4.38
3	0	1	0	1	405.47 ± 6.49
4	−1	0	1	0	325.67 ± 7.52
5	1	0	0	−1	367.81 ± 8.01
6	0	−1	0	1	382.47 ± 6.45
7	−1	1	0	0	344.32 ± 5.69
8	−1	0	0	−1	327.85 ± 4.59
9	1	0	0	1	443.57 ± 7.18
10	1	−1	0	0	377.12 ± 4.09
11	0	0	0	0	425.45 ± 6.18
12	1	0	1	0	371.25 ± 7.42
13	1	0	−1	0	413.25 ± 7.19
14	0	0	0	0	419.26 ± 8.36
15	0	0	1	1	420.24 ± 8.25
16	−1	−1	0	0	319.28 ± 7.19
17	−1	0	−1	0	329.96 ± 6.94
18	0	0	−1	−1	355.49 ± 8.36
19	0	0	1	−1	326.23 ± 7.78
20	0	1	0	−1	336.23 ± 7.26
21	−1	0	0	1	306.45 ± 5.19
22	0	0	−1	1	365.26 ± 6.34
23	0	−1	1	0	321.25 ± 5.77
24	0	1	1	0	366.14 ± 8.06
25	0	−1	−1	0	347.34 ± 4.58
26	0	0	0	0	428.17 ± 5.19
27	0	0	0	0	416.36 ± 6.42
28	0	1	−1	0	381.72 ± 6.35
29	0	0	0	0	407.18 ± 7.18

*Box-Behnken design was used to optimize the conditions for the germination of adzuki beans (Table 1), and a regression model was established as follows. In the model, the effects of four independent variables, namely germination time (A), MSG (B), germination temperature (C), and vacuum time (D), on GABA contents, are represented*.

*Y = 419.28 + 34.98 × A + 10.18 × B – 5.19 × C + 20.44 × D – 4.46 × AB – 9.43 × AC + 24.29 × AD + 2.63 × BC +12.83 × BD + 21.06 × CD – 32.59A^2^ – 30.17B^2^ – 30.97C^2^ – 21.24D^2^*.

**Table 2:** ANOVA in response surface regression model.

**Source**	**Squares**	**df**	**Square**	***F*-Value**	**Prob > *F***	**Significance**
Model	40,819.87	14	2,915.70	13.00	<0.0001	^**^
A	14,686.70	1	14,686.70	65.46	<0.0001	^**^
B	1,244.61	1	1,244.61	5.55	0.0336	^*^
C	322.82	1	322.82	1.44	0.2502	
D	5,014.34	1	5,014.34	22.35	0.0003	^**^
AB	79.39	1	79.39	0.35	0.5614	
AC	355.51	1	355.51	1.58	0.2287	
AD	2,360.02	1	2,360.02	10.52	0.0059	^**^
BC	27.62	1	27.62	0.12	0.7309	
BD	658.44	1	658.44	2.93	0.1087	
CD	1,774.09	1	1,774.09	7.91	0.0138	^*^
A2	6,887.55	1	6,887.55	30.70	<0.0001	^**^
B2	5,902.52	1	5,902.52	26.31	0.0002	^**^
C2	6,221.25	1	6,221.25	27.73	0.0001	^**^
D2	2,926.85	1	2,926.85	13.05	0.0028	^**^
Residual	3,140.85	14	224.35			
Lack of Fit	2,868.81	10	286.88	4.22	0.0890	
Pure Error	272.04	4	68.01			
Cor Total	43,960.72	28				

* *Significant (p < 0.05)*.

** *Extremely significant (p < 0.01)*.

### Response Surface Interaction Analysis

**Figure 3:**
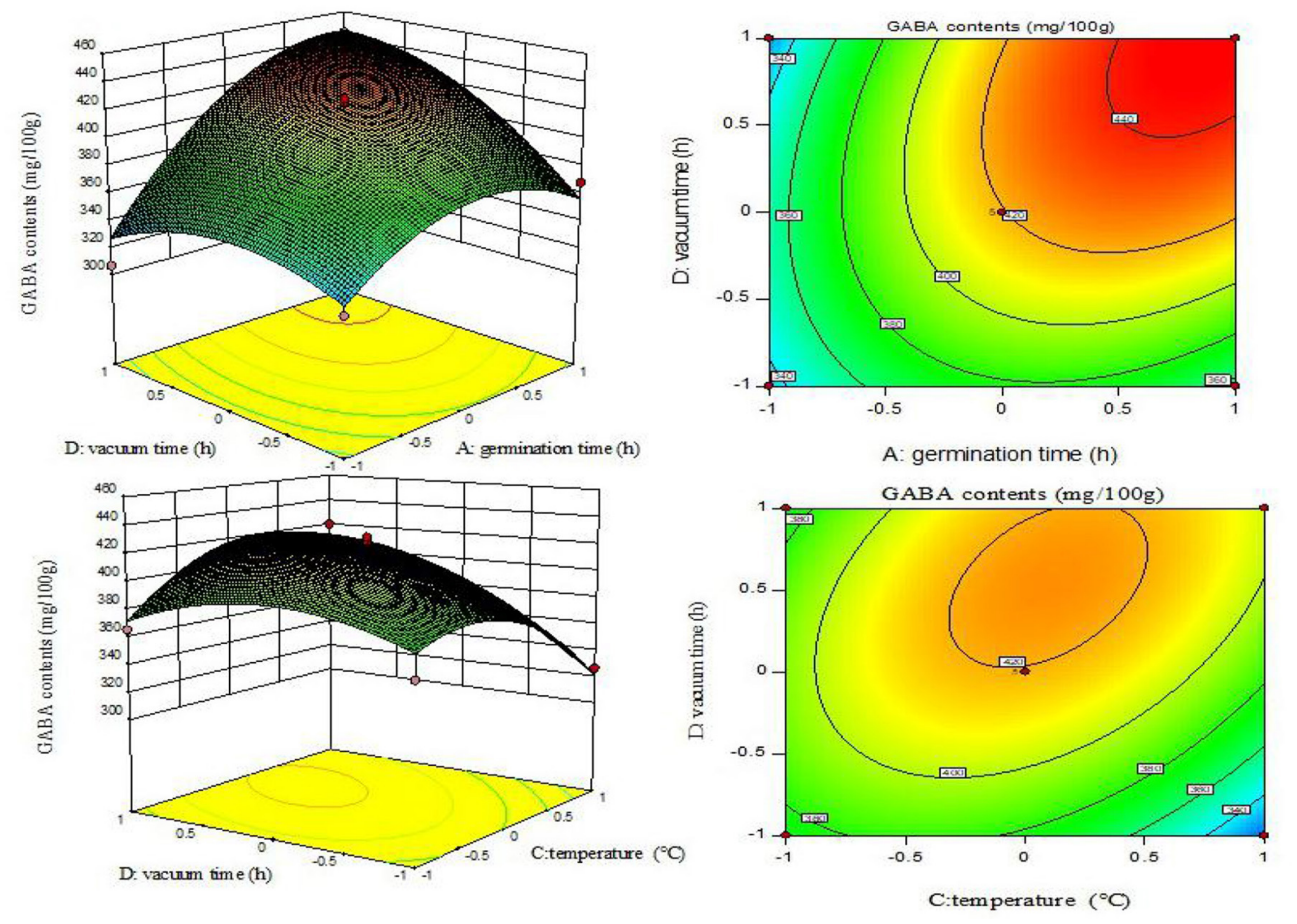
Contour lines and response surface diagrams of the interaction between AD and CD.

The shape of the contour map reflects the interaction effects among various factors (28). Figure 3 presents the response surface and contour plots that had significant interaction effects on the enrichment of GABA contents in adzuki beans.

The AD (germination time and vacuum time) and CD (germination temperature and vacuum time) interactions were significant (*p* < 0.05) (Table 2). The contour map of AD was elliptical (Figure 3), indicating that the interaction between the associated factors was stronger, and the contour map of CD exhibited a gradual slope, indicating a relatively weak interaction between the factors.

### Determination and Verification of Optimal Conditions

After analyses and calculations in Design Expert v8.06, the optimal process parameters for GABA enrichment in adzuki beans were the following: germination time of 48 h, MSG concentrations of 1.99 mg/ml, germination temperature at 31.49°C, and vacuum time of 15.83 h. The predicted GABA contents under these conditions were 443.57 ± 7.18 mg/100 g. Considering the convenience of operation, the actual process parameters were revised to germination time of 48 h, MSG concentrations of 2.0 mg/ml, germination temperature 31°C, and vacuum time of 15 h. There were no significant differences between the predicted and observed experimental data.

### Effects of Enrichment Process on GABA Contents

Control treatment (CK) was a normal germination group, with this as a control, the impact of other treatment methods on GABA enrichment was investigated. The 2 mg/ml MSG treatment (FM) group added an exogenous MSG during germination, providing precursor substances for GABA enrichment, but also a way to increase GABA content. The 15 h vacuum treatment (FZ) group used vacuum processing during germination, hypoxia could decrease the pH of cytoplasm by 0.4–0.8 to create an acidic condition and would produce more GABA to resist the stress; 15 h vacuum + 2 mg/ml MSG treatment (FZM) group first used germination under vacuum to improve the GAD activities. Then, it was subjected to spraying MSG to accumulate more GABA. In addition, the main purpose was to study the changing of enzyme activities and molecular mechanisms during the process of accumulation of GABA in germinating adzuki bean.

**Figure 4:**
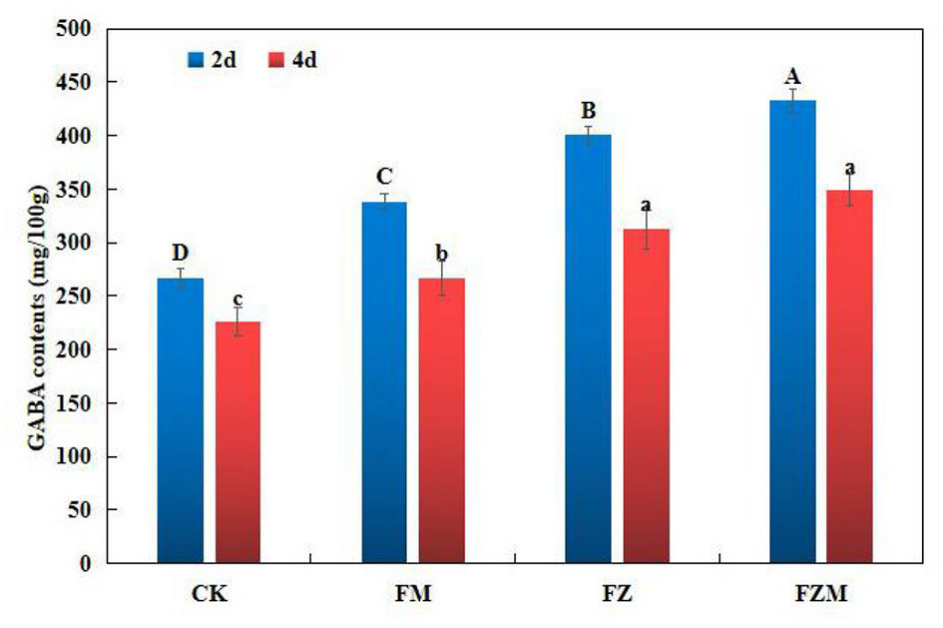
GABA contents in adzuki beans in different germination processes. The soaking conditions of CK, FM, FZ, and FZM were 16 h soaking, soaking temperature at 35°C, and pH value of 5. GABA contents were determined after germination for 2 d and 4 d. Data are mean ± SD (*n* = 3). The upper- and lower-case letters in the figure reflect significant differences between the treatments at 2 and 4 d after germination (*p* < 0.05). CK: Control treatment (31°C for 4 days); FM: CK+2 mg/ml MSG treatment; FZ: CK+15 h vacuum treatment; FZM: CK+15 h vacuum + 2 mg/ml MSG treatment (sprayed every 1 h).

When compared with the contents in the CK treatment, the GABA contents in the FM, FZ, and FZM treatments following 2 days germination increased by 26.73, 50.05, and 62.04%, respectively (Figure 4). In the FZM treatment after 4 days, GABA contents in the 4 days germination seeds were 23.95% lower than those in the 2 days treatment. Therefore, germination under vacuum and MSG treatment would enhance GABA accumulation the most, and the FZM treatment provided contribution to 29.9% of GABA production.

### Changes of GABA Enzyme Activity

**Figure 5:**
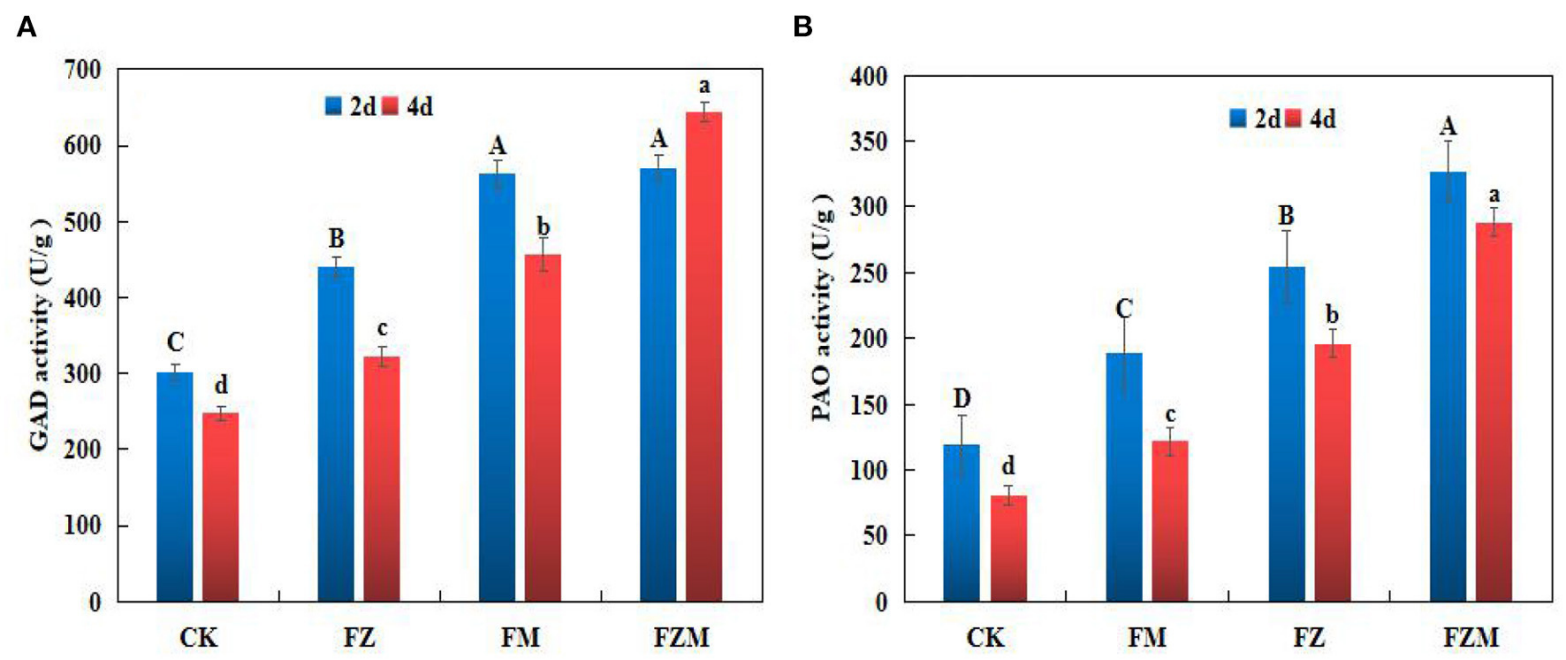
**(A)** Glutamate decarboxylase (GAD) and **(B)** polyamine oxidase (PAO) activity in germinating adzuki beans. Different upper and lower case letters indicate significant differences between the 2 and 4 days germination treatments (*p* < 0.05). CK: Control treatment (31°C for 4 d); FM: 2 mg/ml MSG treatment; FZ: 15 h vacuum treatment; FZM: 15 h vacuum + 2 mg/ml MSG treatment (sprayed every 1 h).

After 2 days germination, GAD activity in the FM, FZ, and FZM treatments increased by 45.75, 86.15, and 89%, respectively, when compared with the activity in CK (Figure 5A), and there was no significant difference between the FZ and FZM treatments. In addition, GAD enzyme activity was significantly higher after 2 days germination than after 4 days germination (*p* < 0.05) in all the treatments.

On the second and fourth day of germination, the FZM had the highest PAO activity at 326.56 and 288.42 U/g prot, respectively, and the activity on day 2 was significantly higher than on day 4 (Figure 5B). The results indicate that FZM (vacuum and MSG treatment) enhanced GAD and PAO activity in germinating adzuki beans. GAD enzyme activity was generally significantly higher than PAO activity.

### GABA Synthase Expression Trends

**Figure 6:**
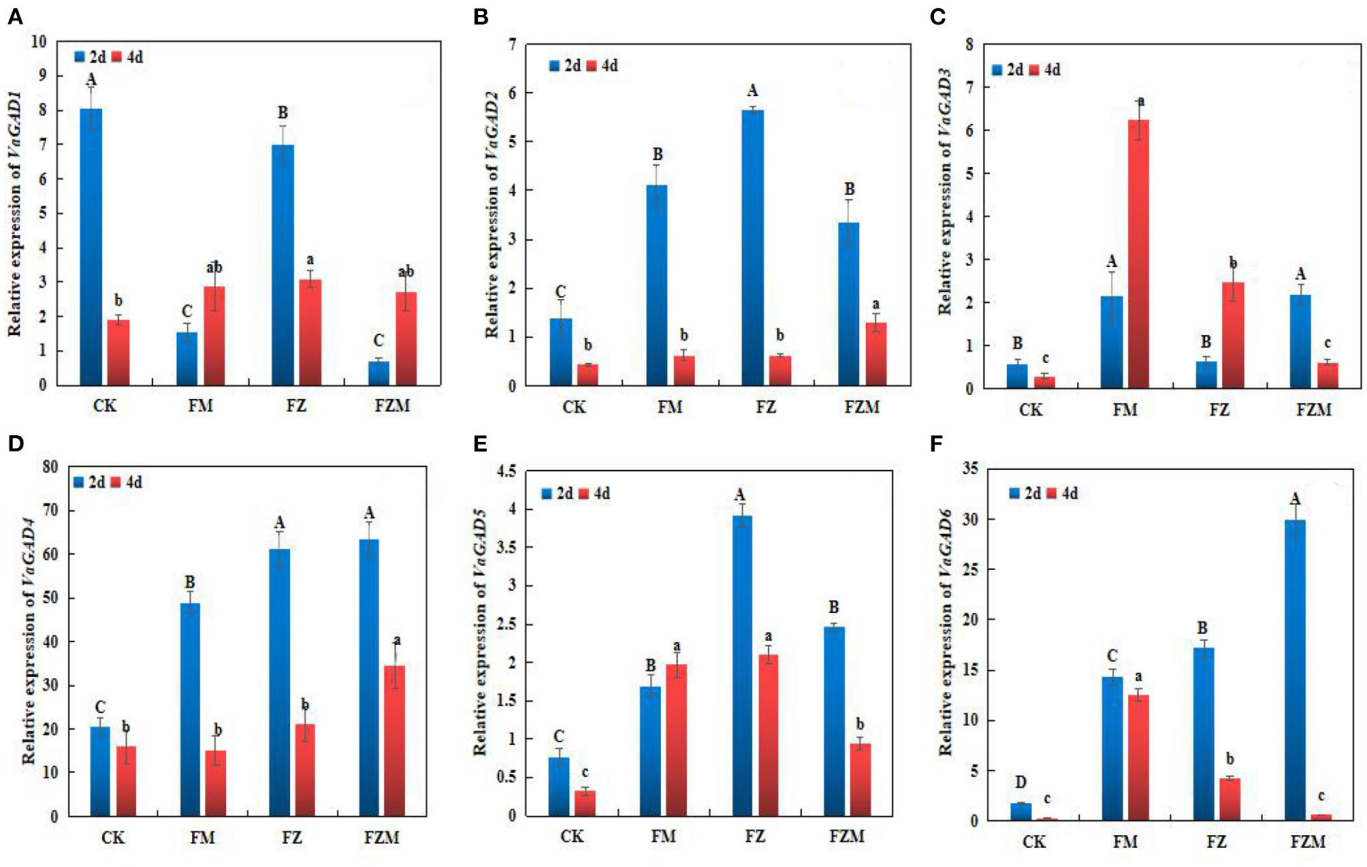
**(A)***VaGAD1*, **(B)***VaGAD2*, **(C)***VaGAD3*, **(D)***VaGAD4*, **(E)***VaGAD5*, and **(F)***VaGAD6* relative expression trends in germinating adzuki beans. The upper and lower case letters in the figure reflect significant differences between treatments at 2 and 4 d of germination (*p* < 0.05). CK: Control treatment (31°C for 4 d); FM: 2 mg/ml MSG treatment; FZ: 15 h vacuum treatment; FZM: 15 h vacuum + 2 mg/ml MSG treatment (sprayed every 1 h).

*VaGAD*-related genes (*VaGAD1–6*) were expressed in different treatment groups (Figure 6). Compared with the control, the FM, FZ, and FZM treatments induced *VaGAD2, VaGAD4, VaGAD5*, and *VaGAD6* expression significantly in 2 days germination beans (*P* < 0.05), and the expression of the four genes decreased at day 4. Under the FZM treatment, among the genes, *VaGAD4* was the most highly expressed at day 2, with ~2.1-, 25.7-, and 28.8-fold higher expression than those of *VaGAD6, VaGAD5*, and *VaGAD2*, respectively, and *VaGAD1* exhibited the lowest expression. *VaGAD5* exhibited increased expression only on the 4th day in the FM treatment, relative to the 2nd day. In contrast, *VaGAD3* expression at day 4 of germination was higher than those at day 2 in the FM and FZ treatments. *VaGAD6* was the most highly expressed at day 4 in the FM treatment, instead, it significantly decreased (*p* < 0.05) in FZ and FZM treatments (Figure 6). Therefore, MSG in combination with vacuum treatment could significantly induce *VaGAD4* and *VaGAD6* expression in 2 days beans. Such expression patterns could have had positive effects on GABA accumulation in 2 days germinating adzuki beans, and GABA contents and synthase activity were consistent with gene expression.

**Figure 7:**
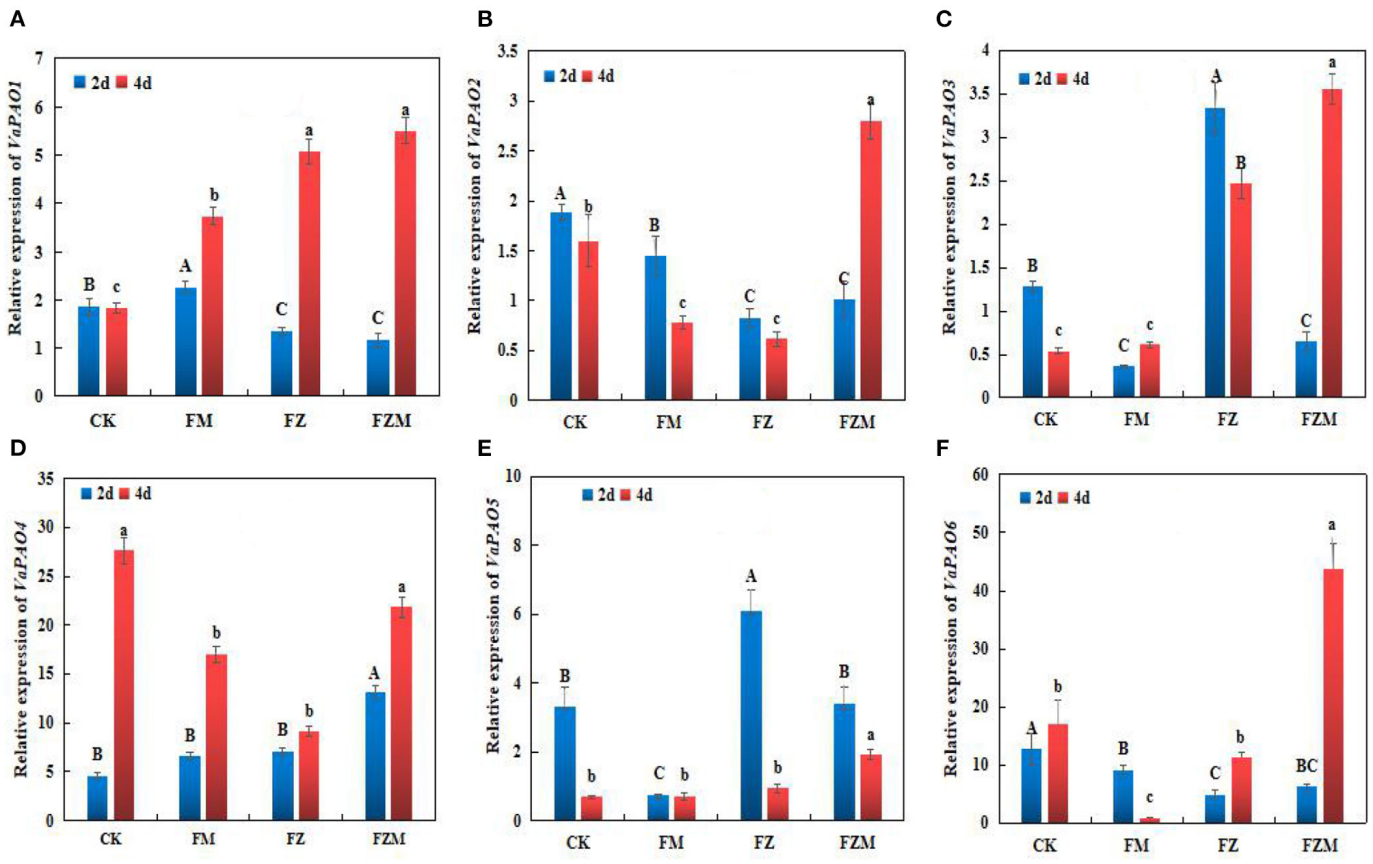
**(A)***VaPAO1*, **(B)***VaPAO2*, **(C)***VaPAO3*, **(D)***VaPAO4*, **(E)***VaPAO5*, and **(F)***VaPAO6* relative expression in germinating adzuki beans. Upper and lower case letters in the figure reflect significant differences between 2 and 4 d germination treatments (*p* < 0.05). CK: Control treatment (31°C for 4 d); FM: 2 mg/ml MSG treatment; FZ: 15 h vacuum treatment; FZM: 15 h vacuum + 2 mg/ml MSG treatment (sprayed every 1 h).

The expression trends of *VaPAO*-related genes in the different germination treatments are illustrated in Figure 7. According to the mean values, the expression levels of *VaPAO4* and *VaPAO6* were higher than those of the other genes in germinating beans, and *VaPAO6* expression was the highest in the FZM treatment, which was almost 3.88-, 50.94-, and 2.58-fold higher than those in the FZ, FM, and CK treatments, respectively. Under the FZM treatment, the expression levels of *VaPAO1, VaPAO2, VaPAO3, VaPAO4*, and *VaPAO6* following 4 days germination were higher than following 2 days germination; however, only *VaPAO5* expression levels decreased.

## Discussion

Immersion of beans and cereals seeds in solution could release dormancy and restore physiological functions. Previous studies have demonstrated that different soaking conditions have varying effects on GABA enrichment in germinating beans (16, 29). In the present study, when the soaking time was <16 h, GABA contents in adzuki beans increased as soaking time increased (Figure 1A), which indicated that metabolism was induced following water imbibition, in addition to the activation of endogenous enzymes and GAD in the reaction system produces GABA secondary metabolites and other nutrients (7, 30). Liao et al. observed that a soaking temperature of 35°C was optimal for the accumulation of GABA contents in adzuki beans (31), which is consistent with the results of the present study.

When the soaking solution pH was 3 and 5, the GABA contents were 81.07 ± 2.31 and 96.53 ± 0.70 mg/100 g, respectively. In addition, compared to soaking temperature, the pH and soaking time had greater impacts on the GABA contents, indicating that the soaking environment influences GABA enrichment, *via* the catalyzation of glutamate decarboxylation with GAD, which is a prerequisite for germination and the optimal pH was generally 5.5–6.0, confirming that an acid culture fluid increases the GABA contents in germinating broad beans significantly, as reported in a previous study (32).

According to the results of this study, exogenous MSG and vacuum treatment are conducive for GABA accumulation. Previous studies have reported that GABA enrichment is associated with adverse conditions, such as mechanical damage, low temperature, hypoxia, and salt stress. Under different stress conditions in the same plant, the rate of increase in GABA contents would be different (16). In this study, 2.0 mg/ml MSG was the optimal spray concentration, with a 1.22-fold increase in GABA contents when compared with the control level.

In this study, vacuum treatment enhanced the GABA contents. Vacuum treatment is a form of hypoxia stress. In hypoxic environments, plant electron transfer chains are inhibited, and the saccharide material is mainly produced by mass alanine *via* glycolysis under such conditions, which leads to the accumulation of ethanol and lactic acid, so that cytokine is acidified. The GAD enzyme of synthetic GABA is conducive to activation, increasing GABA considerably in vacuum-germinated adzuki beans. Using the response surface model (RSM), the optimal treatment conditions for GABA enrichment in adzuki beans were 48 h germination time, 1.99 mg/ml MSG concentration, 31.49°C germination temperature, and 15.83 h vacuum time. The predicted GABA contents under these conditions were 443.57 ± 7.18 mg/100 g.

Liao et al. investigated the effects of hypoxia treatment on fresh tea, and observe increased ammine and spermine expression, CsGAD1 and CsGAD2 expression, and GAD and diamine oxidase (DAO) activity (7). In the study, GABA contents reached 0.73 mg/g FW, which was 20-fold higher than that in the control group. In another study, the GABA content of 5 day fava bean sprout under hypoxia was 2.21-fold higher than that of the control (16). In addition, investigating hypoxia combined with NaCl, CaCl_2_ treatment influenced GABA concentrations in germinating soybean, and the co-treatment group has further improved, and the GABA content improved further compared to only hypoxia treatment, with 4.0 and 3.3 g/kg DW (33). In the study, vacuum and MSG treatment increased GABA content by 1.53-fold, which was 18.54-fold than that of the adzuki beans that did not sprout, further confirming that low oxygen combined with other stresses can enhance GABA enrichment. Therefore, controlling the germination process enriches GABA content naturally, which can be used to increase product applications or improve the health value of the food ingredients (34).

Different factors tested during germination had different levels of contribution to GABA enrichment in adzuki bean, with vacuum and MSG (FZM) treatments having the maximum contribution rate of 29.9% (Figure 4). Furthermore, the FM, FZ, and FZM treatments all increased GABA significantly (*p* < 0.05), with the levels under 2 days germination being greater than at 4 days (Figure 4). The result is consistent with that observed in the case of soybean in a study when low oxygen was combined with freezing (12). Vacuum treatment in combination with MSG treatment could significantly increase GABA contents and the increase was due to an increase in GAD and PAO activity.

During sprouting, different stress treatments induced *VaGAD2, VaGAD4, VaGAD5*, and *VaGAD6* expression significantly in 2 days germination adzuki beans, and the expression of the four genes decreased at day 4, and the FZM treatment could significantly induce *VaGAD4* and *VaGAD6* expression in 2 days germination adzuki beans, which could be essential GAD family genes in germinating adzuki beans. GABA production could be stimulated by an increase in *VaPAO4* and *VaPAO6* expression levels in germinating adzuki beans under vacuum and MSG stress. The results are inconsistent with the PAO activity trends in the germinating adzuki beans. However, whether vacuum and MSG treatment influenced the expression of *VaPAO*-related genes in the present study remains unclear; however, vacuum and MSG stress led to GABA accumulation *via* GAD activation in 2 days germination; simultaneously, the expression of *VaPAO*-related genes increased in 4-days germination. The results suggest that *VaGAD* and *VaPAO*-related genes are essential for GABA synthesis.

Vacuum and MSG treatments induced the expression of some GAD genes in the 2 days germination; which were positively correlated with GABA contents and enzymatic activity. In addition, the expression of *VaPAO*-related genes increased in 4 days germination. The results suggest that *VaGAD* and *VaPAO*-related genes are crucial for GABA synthesis. Optimal expression was observed at 48 h, which is consistent with the 2 days GAD gene expression that also indicated that GABA contents in adzuki beans were enriched. GABA relies mainly on the GABA branch, and the later polyamine degradation pathway plays an important role in GABA enrichment. According to Fang et al. the polyamine degradation route accounts for 39% of the GABA accumulation in soybeans (35).

Similarly, the mechanism of synthesis of GABA in adzuki beans is driven by the dynamic balance of the GABA split and the common effects of the polyamine degradation pathway.

## Conclusion

Soaking and germination conditions influence the enrichment of GABA contents in germinating adzuki bean. The optimal germination conditions were identified as a vacuum in combination with MSG treatment based on germination for 48 h, 1.99 mg/ml MSG concentration, and 41.49°C germination temperature, and 15.83 h vacuum time, with a predicted GABA concentration of 443.57 ± 7.18 mg/100 g, which is 1.53 times the under normal germination, and 18.54 times higher than no germination. Vacuum treatment in combination with MSG treatment (FZM) had the highest contribution to GABA enrichment, at 29.9%, which was associated with increases in GAD and PAO activity. Different stress treatments induced *VaGAD1, VaGAD2, VaGAD4*, and *VaGAD6* expression, and the FZM treatment can be adjacent to PAO; however, PAO activity after 4 days of germination was significantly higher than that after 2 days of germination. In general, *PAO*-related gene expression was lower than *GAD*-related gene expression, indicating that GABA in the pre-bud adzuki bean prior to relying on the GABA branch and the later polyamine degradation pathway had a considerable contribution to the enrichment of GABA contents.

## Data Availability Statement

The raw data supporting the conclusions of this article will be made available by the authors, without undue reservation.

## Author Contributions

DZ and AZ were responsible for the design and overall management of the entire study. YL, LZ, and LG provided the plant lines. JY, HJ, and XS performed the experiments and collected the data. XJ and QX analyzed the data and wrote the manuscript. ZL and DC revised the manuscript. All authors have read and agreed to the publishing of the current version of the manuscript.

## Funding

This work was supported by cooperation research and application demonstration of refined processing key technology of coarse cereal foods (2018YFE0206300) and the evaluation and metabolic mechanism of γ-aminobutyric acid adzuki bean powder (2018YFE0206300-1-12).

## Conflict of Interest

The authors declare that the research was conducted in the absence of any commercial or financial relationships that could be construed as a potential conflict of interest.

## Publisher's Note

All claims expressed in this article are solely those of the authors and do not necessarily represent those of their affiliated organizations, or those of the publisher, the editors and the reviewers. Any product that may be evaluated in this article, or claim that may be made by its manufacturer, is not guaranteed or endorsed by the publisher.
