# Supplementary material for: Optimization of γ-Aminobutyric Acid (GABA) Accumulation in Germinating Adzuki Beans (Vigna angularis) by Vacuum Treatment and Monosodium Glutamate, and the Molecular Mechanisms
Source: Front Nutr. 2021 Sep 9;8:693862. doi: 10.3389/fnut.2021.693862 (PMC8458712; doi:10.3389/fnut.2021.693862)
Supplement: Supplementary file 1 [file Data_Sheet_1.pdf]

Supplementary Figures and Tables

Supplementary table 1. Box-behnken experimental factors and levels

levels	factors			
	A<br>germination time (h)	B<br>MSG（mg/mL）	C<br>temperature(℃)	D<br>vacuum time（h）
-1	24	1.5	25	8
0	36	2.0	30	12
1	48	2.5	35	16

Supplementary table 2. Specific primer sequence for rate-limiting enzyme gene expression in germinated adzuki bean

Primer	Sequences (5'–3')
VaGAD1-F1	ACAATGCCACCAGATGCAGA
VaGAD1-R2	TGGGGCTAGGAAGTGTGTCT
VaGAD2-F	AGTTGATGCTGGATGGCACA
VaGAD2-R	AGCTCAGTGGTGACAGGGTA
VaGAD3-F	AATGGTGTGCCTTTGGTTGC
VaGAD3-R	ATGCTGGCACAATCCATCCA
VaGAD4-F	TGGTTGGGTTATTTGGAGGACA
VaGAD4-R	TCTAAATGTTCCTTCACCACCAT
VaGAD5-F	TGCCAGCTTATCCAATGCCA
VaGAD5-R	CAAACGAAAGGCGCTGAACA
VaGAD6-F	ATTTGGAGAAGAGTGGGCGG
VaGAD6-R	TGGCATTGGATAAGCTGGCA
VaGAD7-F	CAACCTTCGCCTCTCGCTAT
VaGAD7-R	CCAGGTTCAGCCTCGGATTT
VaPAO1-F	CAGGAACATGTCCTCTCGGG
VaPAO1-R	AAGTTCCTGCCATCCTCGAC
VaPAO2-F	AGGGATAGGGTAGGTGGCAG
VaPAO2-R	TTGGTGCCAAGGGATTCTCA
VaPAO3-F	TCAGTGGCAAAAGGGTTCCA
VaPAO3-R	CACCACTTGACCCTACAGCA
VaPAO4-F	GTGCAGAATGGAGGGTTGGT
VaPAO4-R	AGTAGCCCCGGACCATTAGA
VaPAO5-F	GCTGACGAAAGAGGCTACGA
VaPAO5-R	ACCTGGGTAAGCGTGGATTG
VaPAO6-F	GAATGGCAACCCGAGAGACA
VaPAO6-R	CTCCCAGTGAAACCGTGACA
VaPAO7-F	CAAACAGCAGGAGAGGTCCC
VaPAO7-R	ACCCAGGTCAACAGGAAAGC
Actin-F	CTAAGGCTAATCGTGAGAA
Actin-R	CGTAAATAGGAACCGTGT

[1] F: Forward primer sequence, [2] R: Reverse primer sequence
